# Steroid Drugs Inhibit Bacterial Respiratory Oxidases and Are Lethal Toward Methicillin-Resistant *Staphylococcus aureus*

**DOI:** 10.1093/infdis/jiad540

**Published:** 2024-02-13

**Authors:** Samantha A Henry, Calum M Webster, Lindsey N Shaw, Nathanial J Torres, Mary-Elizabeth Jobson, Brendan C Totzke, Jessica K Jackson, Jake E McGreig, Mark N Wass, Gary K Robinson, Mark Shepherd

**Affiliations:** School of Biosciences, University of Kent, Canterbury, United Kingdom; School of Biosciences, University of Kent, Canterbury, United Kingdom; Department of Molecular Biosciences, University of South Florida, Tampa; Department of Molecular Biosciences, University of South Florida, Tampa; Department of Molecular Biosciences, University of South Florida, Tampa; Department of Molecular Biosciences, University of South Florida, Tampa; Department of Molecular Biosciences, University of South Florida, Tampa; School of Biosciences, University of Kent, Canterbury, United Kingdom; School of Biosciences, University of Kent, Canterbury, United Kingdom; School of Biosciences, University of Kent, Canterbury, United Kingdom; School of Biosciences, University of Kent, Canterbury, United Kingdom

**Keywords:** cytochrome *bd*, antimicrobials, drug repurposing, quinestrol, MRSA

## Abstract

**Background:**

Cytochrome *bd* complexes are respiratory oxidases found exclusively in prokaryotes that are important during infection for numerous bacterial pathogens.

**Methods:**

In silico docking was employed to screen approved drugs for their ability to bind to the quinol site of *Escherichia coli* cytochrome *bd*-I. Respiratory inhibition was assessed with oxygen electrodes using membranes isolated from *E. coli* and methicillin-resistant *Staphylococcus aureus* strains expressing single respiratory oxidases (ie, cytochromes *bd*, *bo*′, or *aa*_3_). Growth/viability assays were used to measure bacteriostatic and bactericidal effects.

**Results:**

The steroid drugs ethinylestradiol and quinestrol inhibited *E. coli bd*-I activity with median inhibitory concentration (IC_50_) values of 47 ± 28.9 µg/mL (158 ± 97.2 µM) and 0.2 ± 0.04 µg/mL (0.5 ± 0.1 µM), respectively. Quinestrol inhibited growth of an *E. coli* “*bd*-I only” strain with an IC_50_ of 0.06 ± 0.02 µg/mL (0.2 ± 0.07 µM). Growth of an *S. aureus* “*bd* only” strain was inhibited by quinestrol with an IC_50_ of 2.2 ± 0.43 µg/mL (6.0 ± 1.2 µM). Quinestrol exhibited potent bactericidal effects against *S. aureus* but not *E. coli*.

**Conclusions:**

Quinestrol inhibits cytochrome *bd* in *E. coli* and *S. aureus* membranes and inhibits the growth of both species, yet is only bactericidal toward *S. aureus*.

## Teaser

Computational and microbiological techniques were used to identify the steroid drug quinestrol as an inhibitor of cytochrome *bd* respiratory complexes from *Escherichia coli* and *Staphylococcus aureus*. This drug was bacteriostatic toward both species but was only lethal toward *S. aureus*.

Cytochrome *bd* oxidases, or *bd*-type oxidases, are respiratory oxidoreductases found exclusively in the inner membrane of prokaryotes [1]. The main role of *bd*-type oxidases is to couple quinol oxidation to the reduction of molecular oxygen during which energy is conserved as a proton motive force [2] used in the production of ATP. There are 2 types of *bd*-type oxidase present in *Escherichia coli*: cytochrome *bd*-I and cytochrome *bd*-II [3]. Cytochrome *bd*-I of *E. coli* binds oxygen with high affinity and is expressed under microaerobic conditions whereas cytochrome *bd*-II is expressed under anaerobic conditions [4], and recent work supports the hypothesis that oxygen generation via the decomposition of peroxide provides the electron acceptor for *bd*-II activity during infection [5, 6]. In addition to *bd*-type oxidases, *E. coli* also expresses the cytochrome *bo*′ respiratory oxidase, a heme-copper complex expressed under conditions of high aeration. Two terminal oxidases are expressed by *Staphylococcus aureus*, a cytochrome *aa*_3_ complex (QoxABCD), and a cytochrome *bd* (CydAB) quinol oxidase that is expressed under microaerobic conditions, both of which are important during infection [5].

High-resolution structures exist for a number of cytochrome *bd* complexes [7–9], and several *bd*-type oxidases have been implicated in stress tolerance and virulence in a number of pathogenic bacteria [6, 10–17]. Hence, interest in developing drugs to target *bd*-type oxidases has recently gathered momentum, with antimicrobial peptides/bacteriocins and quinolone derivatives emerging as promising scaffolds (for a detailed review, see [17]). However, thus far there have been no reports of steroid drugs being used to target cytochrome *bd*. Herein, drug repurposing approaches were applied to explore the quinol binding site of cytochrome *bd* as a target for US Food and Administration (FDA)–approved steroid drugs.

## METHODS

### Cytochrome *bd* Modeling and In Silico Drug Screening

**Figure 1.**
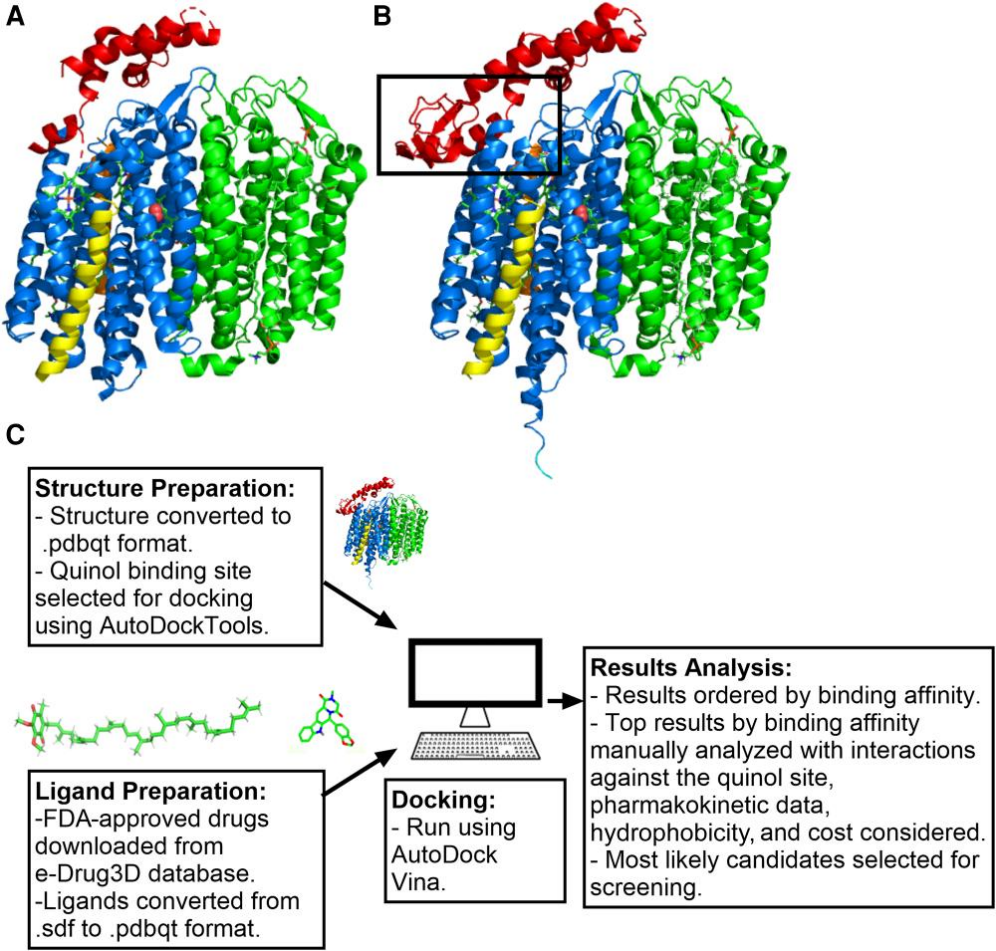
Overview of in silico screening methodology including generation of a structural template for cytochrome *bd*-I. *A*, Published *Escherichia coli* cytochrome *bd*-I structure (PDBid 6RKO). Protein subunits are colored as follows: CydA (blue with Q-loop highlighted in red), CydB (green), CydX (yellow), CydH (orange). Hemes are shown as cylinders (colored by element with carbon = green, nitrogen = blue, and iron = orange), and bound dioxygen is shown as pink spheres. *B*, AF2 model of the *E. coli* cytochrome *bd*-I structure that was used for docking work. The quinol-binding site is highlighted by the black box. *C*, Outline of docking pipeline. Abbreviation: FDA, United States Food and Drug Administration.

The AlphaFold2 protein structure database [18] was used for retrieval of AlphaFold2-modeled *E. coli* K12 CydA (Figure 1*B*). The predicted local distance difference test (pLDDT) scores of model confidence were mostly ranked very high (pLDDT >90). A few regions were ranked confident (90 > pLDDT > 70). These include Leu253, Glu257, Gly238-Val248, and Gln263-Val304 in the Q-loop region. No residues in the Q-loop region had a pLDDT of <70. PyMOL v2.4 was used for structural alignments to create a composite model using AlphaFold2-modeled CydA and the other subunits from PDB structure 6RKO (Figure 1*A*). An *S. aureus* cytochrome *bd* CydA AlphaFold2 model was used for docking to *S. aureus* cytochrome *bd.* The pLDDT scores of model confidence were mostly ranked very high (pLDDT >90), including all the Q-loop apart from Lys277, Thr278, and Glu280, which were ranked confident (90 > pLDDT > 70). All molecular docking work was undertaken using AutoDock Vina [19] (detailed in the Supplementary Methods).

### Bacterial Strains

EC958 is a multidrug-resistant strain of *E. coli* O25: H4-ST131 and the leading cause of urinary tract and bloodstream infections [20–22]. “Cytochrome *bo*′ only” and “*bd*-I only” mutants of *E. coli* were constructed from the wild-type (WT) strain [23] using Lambda-Red mutagenesis [24, 25] (*bd*-only has genotype Δ*cyoA appCB*::Cm, and *bo*′-only has the genotype Δ*cydAB appCB*::Cm). In brief, genes/operons were replaced with chloramphenicol (Cm) resistance cassettes via recombinase expression from the pKOBEG-Gent plasmid [26] and the cassettes were excised via expression of a flippase enzyme encoded on pCP20-Gent [26]. All mutant loci were confirmed via sequencing. The methicillin-resistant *Staphylococcus aureus* (MRSA) strain used is the well-characterized USA300 LAC isolate [27], a community-associated MRSA strain isolated from the Los Angeles County Jail. All MRSA mutant strains were generated by phage transduction of the relevant transposon mutant from the Nebraska Transposon Mutant Library [28] into strain USA300 LAC using methods described previously [29], generating clean insertions in the loci of interest and conferring erythromycin resistance. For the purposes of this article, the MRSA *qoxA::Tn* strain will be referred to as “*bd*-only” while the *cydA::Tn* strain will be referred to as “*aa_3_*-only.”

### Growth Assays

Starter cultures were grown in 10 mL Luria–Bertani (LB) (*E. coli*) or 10 mL Tryptic Soy Broth (TSB) medium (MRSA) in sterile 50 mL conical flasks at 180 rpm and 37°C, until stationary phase was reached, and were used to inoculate 50 mL of fresh growth medium in 250 mL conical flasks. M9 minimal medium was used for *E. coli* (16 g/L Na_2_HPO_4_.2H_2_O, 3 g/L KH_2_PO_4_, 0.5 g/L NaCl, 1 g/L NH_4_Cl, 0.24 g/L MgSO_4_, 0.01 g/L CaCl_2_, 0.1% casamino acids, and 2% glycerol), and TSB medium was used for MRSA. Drug stocks were prepared in dimethyl sulfoxide **(**DMSO) so that their final concentrations were 40× higher than the working concentrations. Greiner F-bottom sterile 96-well plates were prepared by adding 100 µL of a 2× concentrated growth medium, 61.7 µL sterile milliQ H_2_O, 5 µL of the drug, and 33.3 µL of cell culture (final optical density at 600 nm [OD_600_] of ∼ 0.1). Cells were grown in a FLUOstar Omega plate reader at double orbital pattern setting with readings being taken 300 seconds apart. To estimate median inhibitory concentrations (IC_50_), dose-response data were fitted to the sigmoid *y =* Bottom + (Top – Bottom)/(1 + 10^([LogIC_50_ – X]*HillSlope)) equation using nonlinear regression (GraphPad), and standard errors of the fits were calculated.

### Viability Assays

Viability assays were performed essentially as described by Webster et al [22]. In brief, *E. coli* EC958 was grown overnight in 10 mL LB medium and was used to inoculate M9 medium. MRSA cells were grown in TSB medium throughout. Twenty microliters of each drug was added to the wells of row A of a 96-well plate followed by 180 µL of cells (OD_600_ of 0.1). Cells were exposed to drug for 3 hours at 37°C. Following drug exposure, serial dilutions were performed in 1× phosphate-buffered saline (pH 7.4) before being spotted onto LB agar plates overnight to determine changes in cell growth. Six repeats were performed for each concentration of drug, which included 2 biological repeats and 3 technical repeats of each. To estimate median lethal concentrations (LC_50_), dose-response data were fitted to the sigmoid *y* = min + (max – min)/(1 + 10^(n*[logLC_50_ – X])) equation using nonlinear regression (GraphPad), and standard errors of the fits were calculated.

### Membrane Isolation

Cell membranes were isolated as previously described by Poole et al [30]. Cells were grown to exponential phase then harvested at 4000 rpm and 4°C for 20 minutes. The cell pellet was resuspended in ice-cold sonication buffer (20 mM Tris/HCl at pH 7.4, 2 mM MgCl_2_, and 1 mM ethylene glycol-bis(2-aminoethylether)-N,N,N′,N′-tetraacetic acid). The resuspended cells were sonicated (6 × 30 seconds on ice at 15 µM) before centrifugation at 44 000 rpm for 1 hour and 4°C to isolate membranes. The membrane pellet was resuspended in 20 mM Tris/HCl (pH 7.4) at a final concentration of 100 mg/mL and stored at −20°C.

### Oxygen Consumption Assays

Oxygen consumption was measured using a Rank Brothers oxygen electrode as previously described [30] in a 4 mL closed chamber that contained 50 mM HEPES pH 7.4, 0.5 mg/mL membranes (based on wet membranes), and DMSO-solubilized drug (added from 40× final concentration). For *E. coli* membranes, 8 mM succinate (pH 7.4) was added (from 160 mM stock) to initiate the reaction with a single run lasting 15–20 minutes. For MRSA membranes, a final concentration of 500 µM NADH (pH 7.4) was added to start the reaction. Dose-response data were fitted to the sigmoid *y* = min + (max – min)/(1 + 10^(n*[logIC_50_ – X])) using nonlinear regression (GraphPad).

## RESULTS

### Development of a Drug-Docking Pipeline Using FDA-Approved Drugs to Target Cytochrome *bd*

The published *E. coli* cytochrome *bd*-I structure (Figure 1*A*) is missing part of the Q-loop that acts as the quinol binding site (PDB accession number 6RKO [7]). To target the quinol binding site a model is needed to fill in the missing areas, so an AlphaFold2 database model was used to fulfill this purpose (Figure 1*B*). The AlphaFold2 model includes parts of the Q-loop that are missing from the PDB structures, including the conserved N-terminal region between residues Glu240-Val258 and Ala258-Leu310. The CydA model was aligned to the PDB structure 6RKO using PyMOL v2.4, and the other subunits (CydB, CydX, and CydH) were added to create a composite model.

An in silico molecular docking pathway (Figure 1*C*) was set up “in house” using the molecular docking software AutoDock Vina [19] to screen the FDA-approved database for possible inhibitors of cytochrome *bd*-I. The entire FDA-approved library was downloaded from the e-Drug3D online database in 3D.sdf format for a total of 1993 compounds. Structure and ligands were prepared as in Figure 1*C*. Using AutoDockTools, the ligand docking site box coordinates were defined to include just the quinol binding site. FDA ligands with file size above 10 KB were removed (31 in total) as these were deemed unlikely to bind in the quinol binding cleft due to their large size. Ligands with large numbers of flexible bonds were also removed as they greatly reduce the docking accuracy and slowed down the docking process [19]. All of the remaining FDA-approved ligands were then docked to the quinol binding site using AutoDock Vina. The results were analyzed by predicted binding affinity (kcal/mol) (Supplementary Table 1, Figure 1*C*). The top ligands, ordered by predicted affinity, were manually analyzed in PyMOL/LIGPLOT [21, 23] to identify interactions with the key residues in the quinol binding site. MolLogP values as a measure of hydrophobicity were also considered as cytochrome *bd*-I is located within the inner membrane of *E. coli*. Finally, information about known drug function, purchase cost, and pharmacokinetic data was taken into consideration before deciding on which drugs to purchase for laboratory screening. Particular attention was paid to groups of drugs with similar backbone structures that appeared in the top 300 hits, including steroid hormones.

### Docking Steroid Drugs to Cytochrome *bd*-I Quinol Site

**Figure 2.**
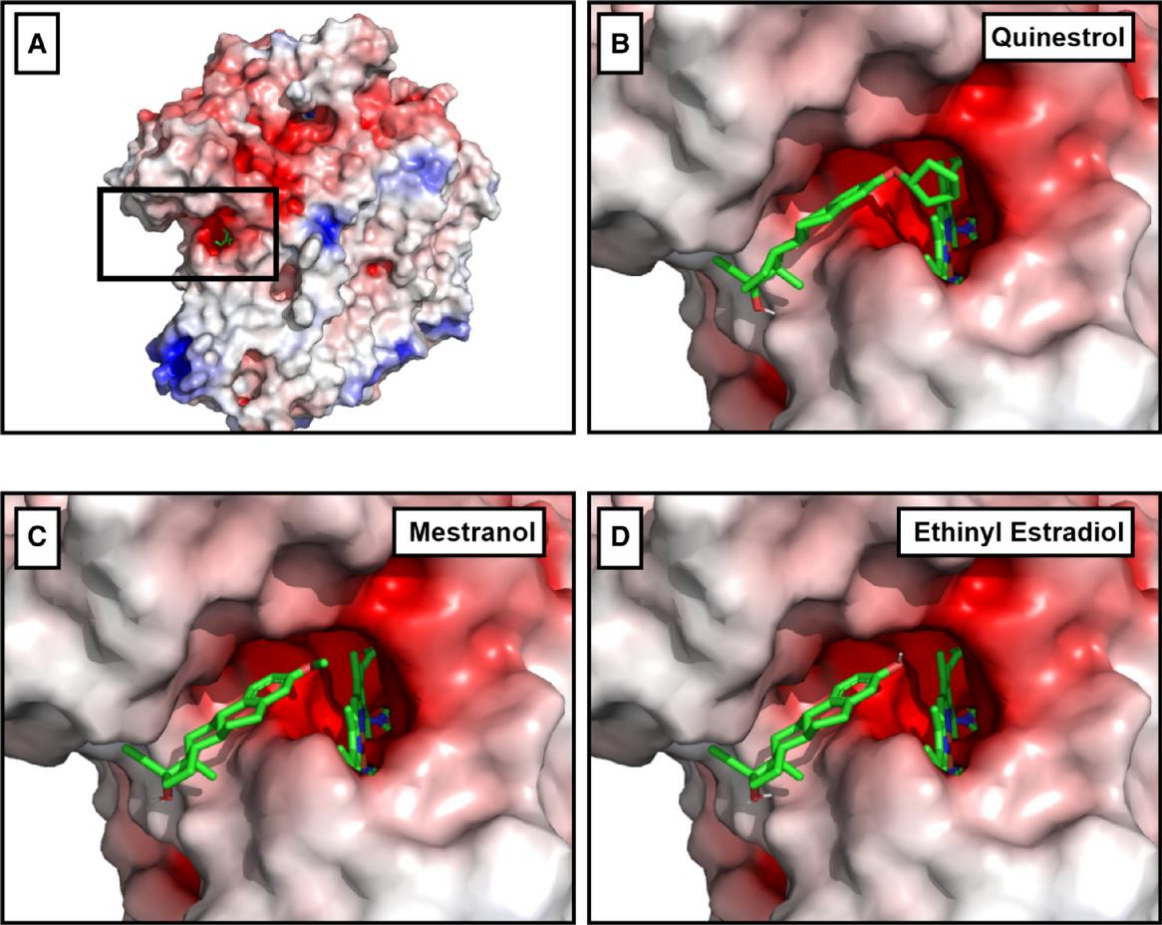
Docking of steroid drugs. *A*, Cytochrome *bd*-I structure with highlighted quinol binding site. *B*, Quinestrol docked in the quinol binding site. *C*, Mestranol docked in the quinol binding site, *D*, Ethinyl estradiol docked in the quinol binding site. Red = negative charge, white = hydrophobic, blue = positive charge.

Several steroid drugs featured in the top hits by predicted binding affinity (Table 1, Supplementary Table 1), including mestranol, quinestrol, and ethinylestradiol (the active metabolite of the prodrug quinestrol). Quinestrol, mestranol, and ethinylestradiol all have the same hydrophobic steroid backbone structure and can dock in a similar configuration in the quinol binding site of *E. coli* cytochrome *bd*-I (Figure 2). All 3 compounds dock with high affinity to the quinol binding site (Table 1) and are hydrophobic, which is a useful property for membrane access. Hence, these were selected for laboratory screening. Quinestrol showed the highest predicted binding affinity to the quinol binding site of the AlphaFold2-modeled structure (−8.6 kcal/mol) and estimated *K*_d_ (489 nM) (Table 1). The steroid drugs mestranol, quinestrol, and ethinylestradiol were also docked onto the *S. aureus* CydA quinol binding site (Supplementary Figure 1). This was using an AlphaFold2 model for *S. aureus* CydA as no PDB structures exist. Quinestrol showed the highest predicted binding affinity to the quinol binding site of the AlphaFold2-modeled structure (−8.1 kcal/mol) and estimated *K*_d_ (1138 nM) (Supplementary Table 2).

**Table 1.** Steroid Compounds and Binding Affinities

Ranking by Predicted Affinity	Drug Name and Structure	e-Drug3D Database Number	Predicted Affinity, kcal/mol	Estimated *K*_d_, nM
39	Quinestrol 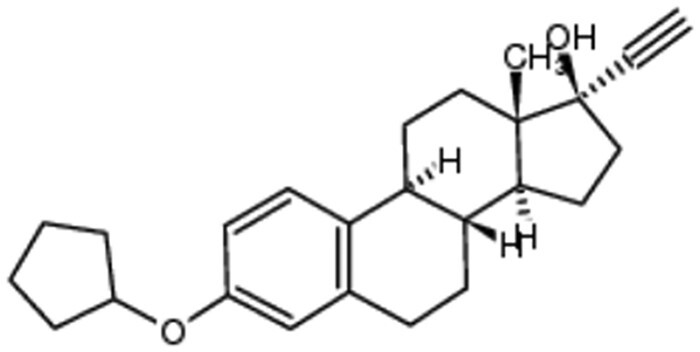	383	−8.6	489
164	Mestranol 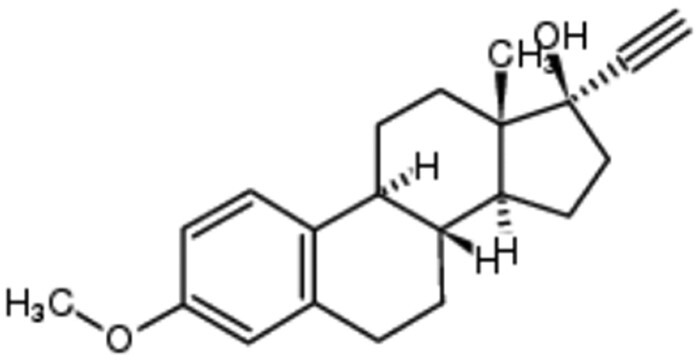	213	−7.9	1595
203	Ethinyl estradiol 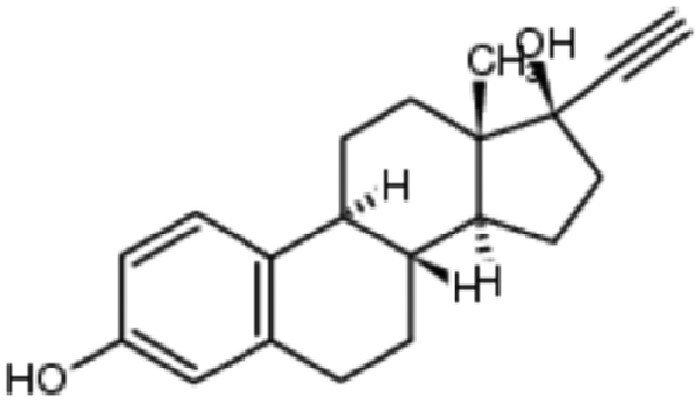	18	−7.8	1889

Steroid drugs were selected based on a combination of binding affinity, hydrophobicity, and modeled interactions with CydA.

### Steroid Drugs Inhibit Oxygen Consumption Activity in *E coli bd*-I-Only Membranes

**Figure 3.**
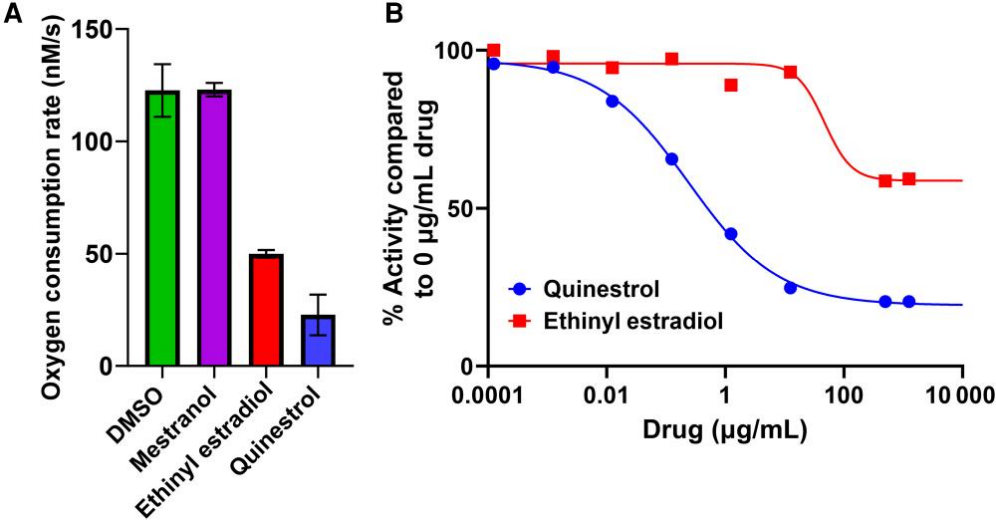
FDA–approved steroid drugs inhibit oxygen consumption activity in *Escherichia coli* EC958 *bd*-I-only membranes. Respiration was initiated by addition of 8 mM succinate as a substrate, and the final concentration of membranes in the reaction chamber was 500 µg/mL. *A*, Initial screening of oxygen consumption using 100 µM drug (dimethyl sulfoxide [DMSO], mestranol, quinestrol, and ethinylestradiol). Drugs were tested in triplicates with error bars representing mean with standard deviation. *B*, Dose-response with quinestrol and ethinylestradiol. Quinestrol exhibited a median inhibitory concentration (IC_50_) of 0.2 ± 0.04 µg/mL (0.5 ± 0.1 µM) whereas ethinylestradiol had an IC_50_ of 47 ± 28.9 µg/mL (158 ± 97.2 µM).

A drug screen was performed to assess the ability of FDA-approved steroid drugs to inhibit the oxygen consumption activity of *E. coli bd*-I only membranes. Initially, 100 µM of each drug was tested to establish whether the steroid drugs had activity against cytochrome *bd*-I function in isolated membranes (Figure 3*A*). Mestranol did not diminish oxygen consumption, whereas both ethinylestradiol and quinestrol inhibited oxygen consumption by 2-fold and 4-fold, respectively (Figure 3*A*). Oxygen consumption measurements demonstrated that varying the concentration of ethinylestradiol and quinestrol has an inhibitory effect on *E. coli bd*-I-only membranes (Supplementary Figure 2), with dose-response curves for ethinylestradiol and quinestrol resulting in IC_50_ values of 47 ± 28.9 µg/mL (158 ± 97.2 µM) and 0.2 ± 0.04 µg/mL (0.5 ± 0.1 µM), respectively. This identified quinestrol as the most potent of the steroid drugs tested and was the focus of the remaining research. Kinetic assays confirmed that quinestrol does not inhibit succinate dehydrogenase activity in *E. coli bd*-I-only membranes and is likely targeting cytochrome *bd*-I (Supplementary Figure 3).

### Quinestrol Suppresses the Growth of *E coli* and MRSA

**Figure 4.**
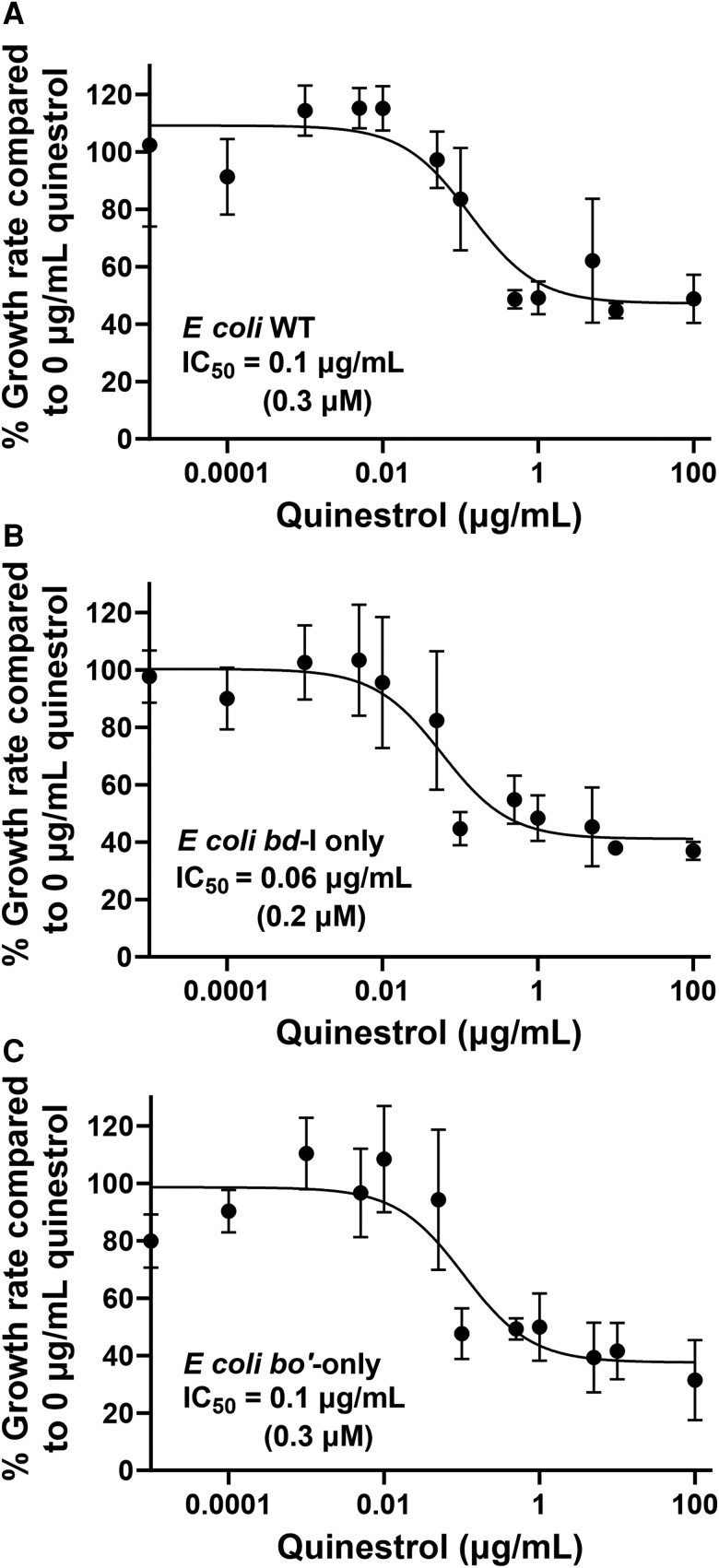
Quinestrol inhibits the growth of *Escherichia coli*. Six repeats were performed for each drug concentration and are represented by the error bars showing mean with standard deviations. *A*, *Escherichia coli* wild-type (WT) cells are inhibited by quinestrol at a median inhibitory concentration (IC_50_) of 0.1 ± 0.02 µg/mL (0.3 ± 0.06 µM). *B*, *Escherichia coli bd*-I-only cells are inhibited by quinestrol with an IC_50_ of 0.06 ± 0.02 µg/mL (0.2 ± 0.07 µM). *C*, *Escherichia coli bo*′-only cells are inhibited by quinestrol at an IC_50_ of 0.1 ± 0.01 µg/mL (0.3 ± 0.03 µM).

It was important to confirm that heme cofactors were properly incorporated into cytochrome *bd*-I before growth experiments were undertaken, so whole-cell CO difference spectra for *E. coli* EC958 WT, *bd*-I-only, and *bo*′-only cells were recorded (Supplementary Figure 4). Peaks and troughs at 640 nm and 620 nm, respectively, confirmed the correct assembly of cytochrome *bd*-I in WT and *bd*-I-only cells. Growth curves were recorded in the presence of varying concentrations of quinestrol, and representative raw data are shown in Supplementary Figure 5 to demonstrate how growth rates were calculated from the windows of maximum growth. The resultant dose-response data for *E. coli* EC958 WT, *bd*-I-only, and *bo*′-only strains are shown in Figure 4. Experiments with WT and *bo*′-only strains produced IC_50_ values of 0.1 ± 0.02 µg/mL (0.3 ± 0.06 µM) and 0.1 ± 0.01 µg/mL (0.3 ± 0.03 µM), respectively (Figure 4*A*, Figure 4*C*). Data for the strain expressing cytochrome *bd*-I as the sole respiratory oxidase (ie, *bd*-I-only) produced a marginally lower IC_50_ of 0.06 ± 0.02 µg/mL (0.2 ± 0.07 µM) (Figure 4*B*), although the errors of the fits suggest that there is no difference between all 3 IC_50_ values for these *E. coli* strains.

**Figure 5.**
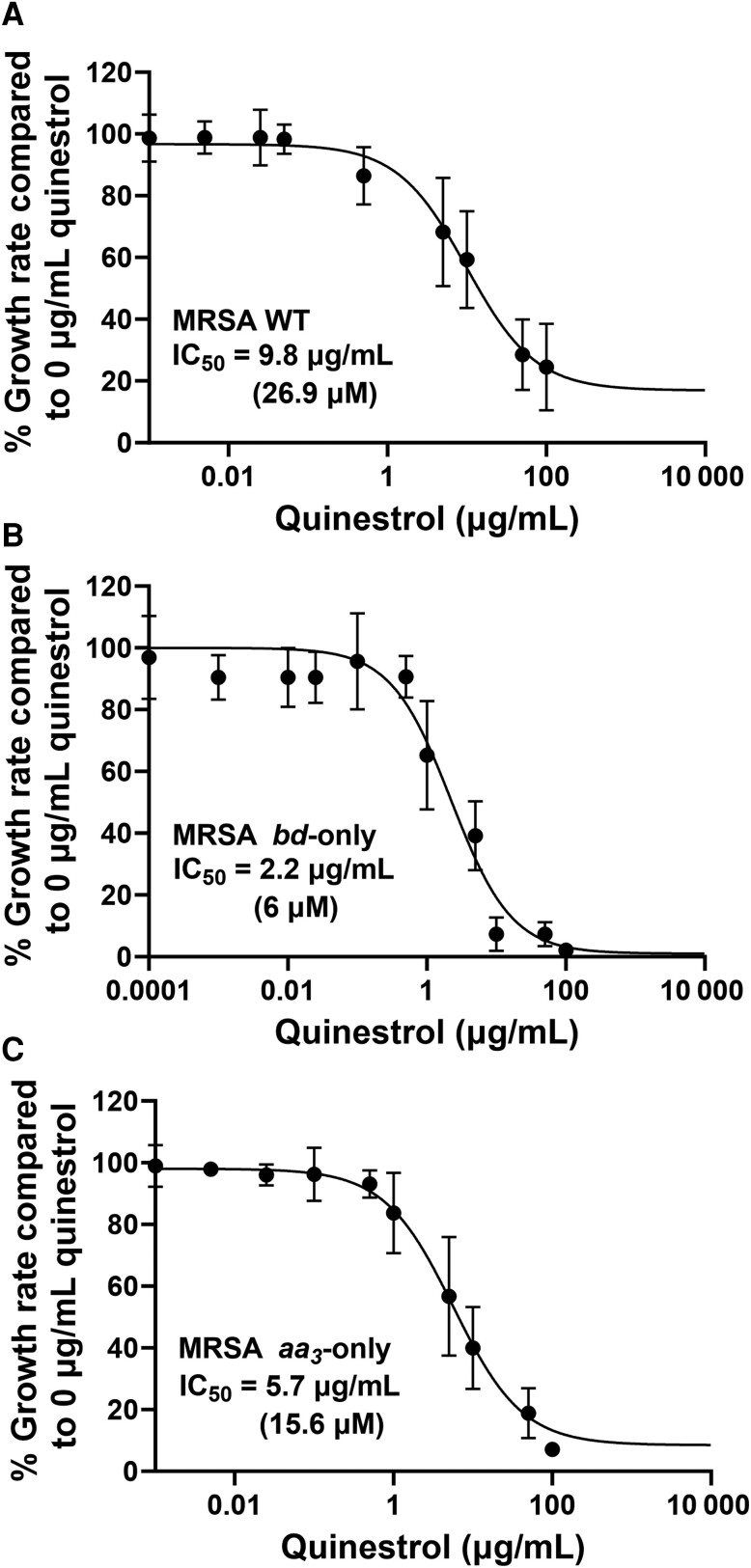
Quinestrol inhibits the growth of methicillin-resistant *Staphylococcus aureus* (MRSA). Six repeats were done for each drug concentration and are represented by the error bars showing mean with standard deviations. *A*, Quinestrol inhibits the growth of wild-type (WT) MRSA cells with a median inhibitory concentration (IC_50_) of 9.8 ± 1.95 µg/mL (27.0 ± 5.4 µM). *B*, Quinestrol inhibits growth of MRSA *bd*-only cells with an IC_50_ of 2.2 ± 0.43 µg/mL (6.0 ± 1.2 µM). *C*, Quinestrol inhibits MRSA *aa_3_*-only cells with an IC_50_ of 5.7 ± 1.2 µg/mL (15.6 ± 3.3 µM).

To investigate the activity of quinestrol against a gram-positive pathogen, growth inhibition assays were conducted on WT, *bd*-only, and *aa_3_*-only strains of MRSA USA300. Growth of all strains was inhibited by quinestrol (Figure 5), and data from the *bd*-only experiments produced a significantly lower IC_50_ of 2.2 ± 0.43 µg/mL (6.0 ± 1.2 µM). Further analyses confirmed that quinestrol inhibited oxygen consumption of MRSA *bd*-only membranes (Supplementary Figure 6).

### Quinestrol Is Bactericidal Against MRSA but Not *E coli*

**Figure 6.**
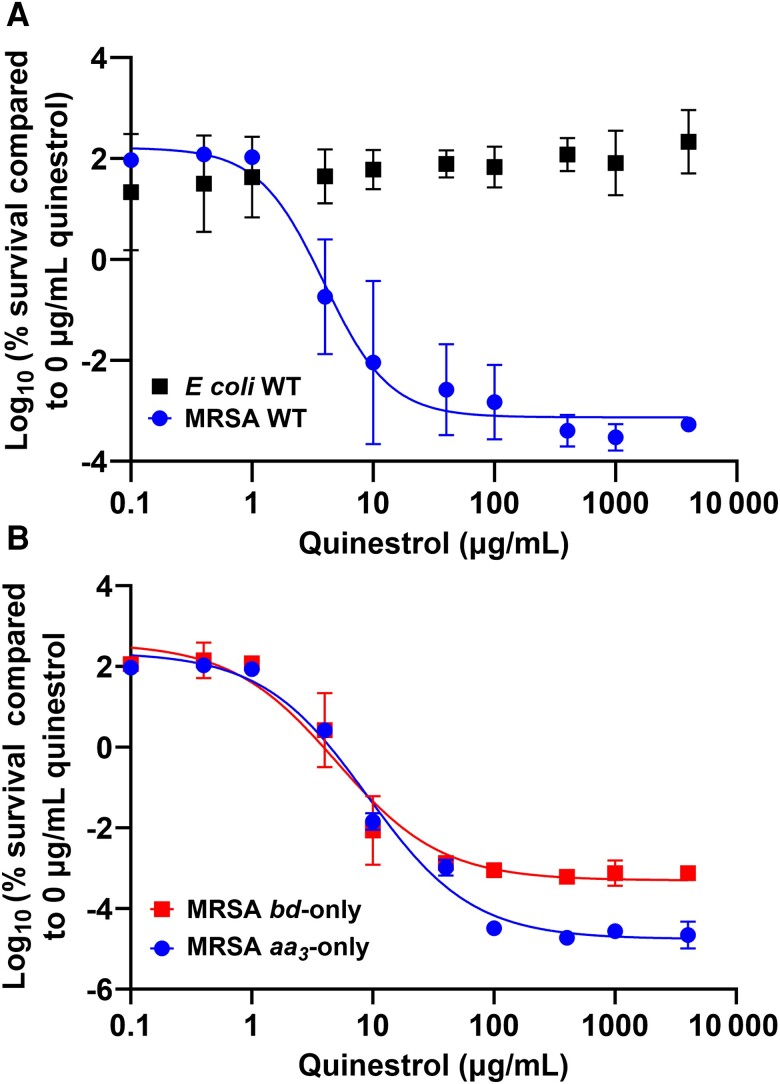
Quinestrol kills methicillin-resistant *Staphylococcus aureus* (MRSA) cells but not *Escherichia coli*. Six repeats were recorded for each drug concentration, and data points show the mean with error bars showing standard deviations. *A*, MRSA wild-type (WT) cells are killed by quinestrol at a median lethal concentration (LC_50_) of 3.4 ± 0.7 µg/mL (9.3 ± 1.9 µM). Quinestrol is not lethal toward *E. coli* WT cells. *B*, Quinestrol kills MRSA *bd*-only cells with an LC_50_ of 5.6 ± 0.3 µg/mL (13.7 ± 0.7 µM). MRSA *aa_3_*-only cells are killed with an LC_50_ of 9.0 ± 0.6 µg/mL (24.7 ± 1.6 µM).

To measure the bactericidal activity of quinestrol, viability assays were performed on *E. coli* and MRSA WT strains. While quinestrol did not kill *E. coli* WT cells, MRSA WT cells were killed by quinestrol with a LC_50_ of 3.4 ± 0.7 µg/mL (9.3 ± 1.9 µM) (Figure 6*A*). Further work on *E. coli* mutants revealed that quinestrol had no killing effects on *bd*-I-only or *bo*′-only cells (data not shown), although quinestrol killed *bd*-only and *aa_3_*-only strains of MRSA with LC_50_ values of 5.6 ± 0.3 µg/mL (13.7 ± 0.7 µM) and 9.0 ± 0.6 µg/mL (24.7 ± 1.6 µM), respectively (Figure 6*B*).

## DISCUSSION

Our in silico drug screen generated a list of FDA-approved drugs with high binding affinities for the quinol site of an AlphaFold2 model of a *E. coli bd*-I structure, which identified steroid drugs as promising candidates for binding to the quinol site. Subsequent in vitro analyses demonstrated that ethinylestradiol and quinestrol inhibited *E. coli bd*-I-only membranes to varying degrees whereas mestranol exhibited no inhibitory effects, which suggested that subtle structural variations could elicit changes in binding affinity. The 3 steroids used in the current study differ only via the substituent on the C3 atom of the A ring: Ethinylestradiol has a hydroxyl group, mestranol has a methyl ether, and quinestrol has a cyclopentyl ether. Together, our findings are consistent with the hypothesis that the A ring is in close proximity to the heme *b*_558_ cofactor, and more hydrophobic substituents (ie, cytopentyl or methyl moieties) promote binding deep in the quinol pocket.

Further analysis revealed the IC_50_ of quinestrol for inhibiting oxygen consumption in *E. coli bd*-I-only membranes as 0.2 ± 0.02 µg/mL (0.5 µM ± 0.04 µM), although residual activity remained at around 20% at higher concentrations (Figure 3*B*). This residual activity suggests that quinestrol is unable to completely inhibit *E. coli* cytochrome *bd*-I terminal oxidase and further kinetic analyses will be required to elucidate the precise modes of binding. Indeed, this trend of low IC_50_ with incomplete inhibition is mirrored by the growth assays for all the *E. coli* strains (Figure 4). Subsequent growth inhibition work for the *bd*-only mutant strain of MRSA produced an IC_50_ value in the low micromolar range (Figure 5*B*), and these data showed no residual growth, suggesting that quinestrol is a more effective bactericidal agent against MRSA. The MRSA WT and *aa*_3_-only strains exhibited IC_50_ values slightly higher than the *bd*-only strain, suggesting that quinestrol binds to *bd* slightly more tightly than *aa*_3_. That said, quinestrol is clearly also a potent inhibitor of the cytochrome *aa*_3_ complex as evidenced by a the *aa*_3_-only strain having a lower IC_50_ compared to the WT strain.

Stark differences were observed between *E. coli* and MRSA when bactericidal activity of quinestrol was assessed, where *E. coli* was completely resistant to killing and all MRSA strains exhibited several log-fold reductions in viability with LC_50_ values in the low micromolar range. This is consistent with a previous screen where quinestrol was shown to inhibit the growth of vancomycin-resistant *Enterococcus faecium* and MRSA but not gram-negative species [31], and perhaps reflects the suitability of steroid-based drugs for targeting bacterial species such as MRSA that lack an outer membrane. Surprisingly, the LC_50_ for the MRSA WT strain is marginally lower than the IC_50_ for this strain, even though cell density was the same upon quinestrol exposure, although this difference in susceptibility could potentially be explained by the metabolic state of the cells being different in the static viability assays (less active) compared to the growth assays where orbital aeration may increase respiratory metabolism (more active). Indeed, it is well-known that some antibiotics target actively growing cells more effectively while others target less metabolically active cells [32]. However, it is difficult to interpret the observed patterns of IC_50_ and LC_50_ between the WT and mutant MRSA strains, as loss of respiratory oxidases appears to diminish the lethality of quinestrol (ie, increases LC_50_) yet enhances the quinestrol-mediated growth inhibition (ie, decreases IC_50_). This could perhaps be linked to differences in metabolic activity between strains and experimental conditions, but it would be too speculative at this time to comment further on the bacteriostatic and bactericidal mechanisms. Notwithstanding these elusive insights, robust conclusions can be made: Quinestrol can inhibit both *bd*-type and heme-copper oxidases and is lethal toward MRSA cells.

Cytochrome *bd* complexes are restricted to the prokaryotic world, which is an attractive trait when selecting potential drug targets. A small number of cationic amphiphilic peptides have been identified that bind to *E. coli* cytochrome *bd*-I, including gramicidin S [33], microcin J25 [34], and cathelicidin LL-37 [35]. While these have IC_50_ values in the µM range, they have complex mechanisms of action thought to primarily involve membrane destabilization rather than direct targeting of the *bd* complex. Cytochrome *bd* from *Mycobacterium tuberculosis* has attracted much attention as a promising target for next-generation antibacterials (recently reviewed in [17, 36]). Bedaquiline, an FDA-approved treatment for tuberculosis and inhibitor of mycobacterial ATP synthases, has been found to increase in efficacy when cytochrome *bd* has been knocked out [37], highlighting future combinatorial treatments alongside cytochrome *bd* inhibitors as promising new therapies. Isoniazid, a first-line treatment for tuberculosis, works via a complex mechanism that involves perturbation of the respiratory chain, and loss of cytochrome *bd* synthesis [38] or inhibition with aurachin C [39] enhances the efficacy of isoniazid. Furthermore, a recent in silico screen identified an inhibitor (MQL-H_2_: 3-[[2-(4-chlorophenyl)ethylamino]methyl]-1-ethyl-indole-2-carboxylic) that binds to the menaquinol-binding pocket of *M. tuberculosis* cytochrome *bd*, and efficacy was tested using ATP assays on *Mycobacterium smegmatis* mutants, revealing an IC_50_ of 34 µM toward a *bd*-only strain [40]). A subsequent study identified a series of 2-aryl-quinolone inhibitors that target *M. tuberculosis* cytochrome *bd* [2], and a very low IC_50_ of 3 nM was calculated for the CK-2-63 compound from inhibition kinetic data for the purified recombinant *M. tuberculosis* cytochrome *bd* isolated from an *E. coli* respiratory mutant strain. Finally, in silico screening of FDA-approved drugs docking to the *Geobacillus thermodenitrificans* cytochrome *bd* structure and subsequent efficacy assays led to the development of the synthetic compound 8d from the quinoline moiety of ivacaftor and roquinimex [41]. This “8d” compound was shown to bind to purified mycobacterial cytochrome *bd* with a *K*_d_ value of 4.17 μM and to inhibit a *bd*-only strain of *M. smegmatis* with a minimum inhibitory concentration value of 6.25 μM. These recent studies highlight the potential for targeting *bd*-type oxidases using derivatives and mimics of natural quinones found in bacteria, which differs from the current study that highlights the novel utility of steroid drugs as respiratory inhibitors.

## CONCLUSIONS

This study demonstrates that steroids are a promising scaffold for future drug development and are effective at targeting multiple ubiquinone-binding and menaquinone-binding *bd*-type and heme-copper oxidases, and appear to be particularly effective against MRSA. Whereas this sort of promiscuity is unwelcome for off-target effects in humans, this is clearly not an issue for these FDA-approved drugs, and multiple sites of action in bacterial pathogens are of obvious benefit. However, interventions would have to be taken to control the catabolism and transport/sequestration of such a drug in vivo to improve the pharmacokinetic properties if this class is to become suitable for the treatment of chronic or deep-seated infections, although other applications (eg, topical) may be of interest in future to combat cutaneous infections.

## Supplementary Data

Supplementary materials are available at *The Journal of Infectious Diseases* online (http://jid.oxfordjournals.org/). Supplementary materials consist of data provided by the author that are published to benefit the reader. The posted materials are not copyedited. The contents of all supplementary data are the sole responsibility of the authors. Questions or messages regarding errors should be addressed to the author.

## Supplementary Material

jiad540_Supplementary_Data

## Notes

***Author contributions.*** All authors contributed to writing the manuscript. S. A. H. was involved in planning and performing most of the microbiological experiments and data analysis. C. M. W. was involved in planning and performing the in silico experiments and data analysis. L. N. S. provided insights and resources for the MRSA work. N. J. T., M.-E. J., B. C. T., and J. K. J. performed the phage transduction work. J. E. M. contributed to setting up the in silico pipeline. M. N. W. contributed to designing the bioinformatics pipeline. G. K. R. contributed to the planning of the microbiological experiments and data analysis. M. S. was responsible for input to all experimental design and data analysis.

***Acknowledgments*.** We thank the Kent Fungal Group for access to the FLUOstar Omega plate reader.

***Ethical approval.*** No ethical approval was required for this study.

***Financial support*.** This study was partially funded by a VC PhD studentship (University of Kent) and by the National Institute of Allergy and Infectious Diseases **(**grant numbers AI124458 and AI157506 to L. N. S.).
