# Supplementary material for: Steroid Drugs Inhibit Bacterial Respiratory Oxidases and Are Lethal Toward Methicillin-Resistant Staphylococcus aureus
Source: J Infect Dis. 2024 Feb 13;230(1):e149–58. doi: 10.1093/infdis/jiad540 (PMC11272085; doi:10.1093/infdis/jiad540)
Supplement: jiad540_Supplementary_Data [file jiad540_supplementary_data.docx]

**SUPPLEMENTARY MATERIAL**

**SUPPLEMENTARY METHODS**

***In silico* drug docking**

Preparation of *in silico* ligand and protein files was performed using AutoDockTools [20] and PyMOL [1]. FDA-approved drugs from the e-Drug3D online database (all FDA approved with molecular weight ≤2000 g/mol) [2] were downloaded in 3D .sdf format). These were converted into .pdb format using PyMOL and then onto a flexible (.pdbqt) format using python script (prepare_ligand4.py) from MGLTools [3]. The cytochrome *bd*-I input structure for molecular docking had the polar hydrogens added using PyMOL. Ligands below 10KB file size were selected (i.e. molecules small enough to dock in the quinol cleft) and docked to the quinol binding site of the *E. coli* cytochrome *bd*-I AlphaFold2 model using Autodock Vina. Out of a total of 1993 ligands 31 were removed from the analysis based on size with each having a molecular weight greater than 1000 Da. Docking of known inhibitors was performed to identify the region known to bind small molecules, which was then used to focus the docking of the FDA-approved drugs. The docking region included the residues Ala230-Asp247, Leu253-Ala258, Ala268-Pro275, Lys283-Val300 and Val383-Ile403. A Bash script was used for automation of the ligand docking.

AutoDock Vina docking results were first analysed by predicted binding affinity (kcal/mol). A python script extracted the binding affinity for the top docking pose for each ligand and ranked them in order of binding affinity. For known inhibitors the equation *K*_d_ = exp(∆G/(R*T) with ∆G = binding energy (kcal/mol), R = gas constant = (1.986 cal/mol*K) and T = temperature (298K) was used to estimate the binding *K*_d_. The top ligands by binding affinity were manually analysed in PyMOL looking for specific interactions to the quinol binding site. LIGPLOT v 4.5.3 was also used to analyse the binding site interactions [4]. Hydrophobicity taken from the MolLogP values, known drug function, and pharmacokinetic data available from e-Drug3D [2] and PubChem [25] databases were all considered before deciding on which compounds to purchase for experimental screening.

The AlphaFold 2 CydA model for *S. aureus* was used to dock the steroid drugs mestranol, quinestrol and ethinylestradiol to the quinol binding site using the same method as for *E. coli* cytochrome *bd*-I.

**Succinate dehydrogenase assays**

The method used for the succinate dehydrogenase assay is an adaptation of Kolaj-Robin et al. 2011 [6]. A 1 mL mixture was prepared with a final concentration of 400 μM phenazine methosulfate (PMS), 8 mM succinate, 50 mM Tris-HCL (pH 8.5) buffer, 0.02 % n-dodecyl-β-D-maltoside, 2.5% drug, 500 µg/mL of bacteria membranes and 50 μM of dichlorophenolindophenol (added last). The phenazine methosulfate, succinate and membranes were first preincubated for 30 min at 37 °C before being added to the remaining mixture. To start the reaction, 50 μM of dichlorophenolindophenol was added that produced a starting OD_600_ that ranged from 0.8 to 1. Spectrakinetics were recorded on a Cary 60 spectrophotometer for 15 min.

**CO difference spectra**

A SDB Spectrophotometer Instrument [7] was used to record the ‘CO difference’ spectra (‘carbon monoxide reduced’ *minus* ‘reduced’) of WT, ‘*bd-*I only’ and ‘*bo*′ only’ *E. coli* cells essentially as described in Poole et al. 1989 [8]. Firstly, 100 mL LB cultures were grown in 250 mL conical flasks (37°C, 130 rpm) until stationary phase and harvested at 4000 rpm for 10 min at 4 ℃. Pellets were resuspended in 4-6 mL of 20 mM Tris-HCl (pH 7.4) and kept on ice. Final OD_600_ of cell suspensions were in the range of 40-60. A few grains of sodium dithionite were added to cell suspensions to produce the reduced sample, and the reduced cell suspensions were later bubbled with carbon monoxide gas for 5 min to generate the ‘CO reduced’ samples. Absorbance spectra were recorded for all samples from 400-700 nm at 0.5 nm/s, and baseline subtractions were performed to generate the difference spectra.

**REFERENCES**

1. Seeliger D, Groot BL De. Ligand docking and binding site analysis with PyMOL and Autodock/Vina. J Comput Aided Mol Des **2010**; 24:417–422.

2. Pihan E, Colliandre L, Guichou JF, Douguet D. E-Drug3D: 3D structure collections dedicated to drug repurposing and fragment-based drug design. Bioinformatics **2012**; 28:1540–1541.

3. Morris GM, Ruth H, Lindstrom W, et al. AutoDock4 and AutoDockTools4: Automated docking with selective receptor flexibility. J Comput Chem **2009**; 30:2785–2791.

4. Wallace AC, Laskowski RA, Thornton JM. LIGPLOT: a program to generate schematic diagrams of protein-ligand interactions. Protein Eng **1995**; 8:127–134.

5. Kim S. Getting the most out of PubChem for virtual screening. Expert Opin Drug Discov **2016**; 11:843–855.

6. Kolaj-Robin O, O’Kane SR, Nitschke W, Léger C, Baymann F, Soulimane T. Biochemical and biophysical characterization of succinate: Quinone reductase from *Thermus thermophilus*. Biochim Biophys Acta Bioenerg **2011**; 1807:68–79.

7. Poole RK, Kalnenieks U. Introduction to light absorption: visible and ultraviolet spectra. Spectrophotometry and Spectrofluorimetry. Oxford University Press **2000**; 1-32.

8. Poole RK, Williams HD, Downie JA, Gibson F. Mutations affecting the cytochrome *d*-containing oxidase complex of *Escherichia coli* K12: identification and mapping of a fourth locus, *cydD*. J Gen Microbiol **1989**; 135:1865–1874.

**SUPPLEMENTARY DATA**

**Supplementary Table S1. Top 70 potential *E. coli* cytochrome *bd*-I inhibitors ranked by predicted affinity to the quinol binding site of the AlphaFold 2 modelled structure.** Drugs of particular interest in bold were tested experimentally. Mestranol and ethinyl estradiol also included for reference.

| **Ranking by predicted affinity** | **Drug Name** | **eDrug3D Database Number** | **Predicted Affinity (kcal/mol)** | **Estimated**  ***K*_d_ (nM)** |
| --- | --- | --- | --- | --- |
| 1 | LOMITAPIDE | 1572 | -9.8 | 64 |
| 2 | LEDIPASVIR | 1731 | -9.8 | 64 |
| 3 | ERGOTAMINE | 70 | -9.6 | 90 |
| 4 | IRINOTECAN | 755 | -9.4 | 127 |
| 5 | NILOTINIB | 1310 | -9.4 | 127 |
| 6 | CALCIPOTRIENE | 1412 | -9.4 | 127 |
| 7 | DOXERCALCIFEROL | 1417 | -9.4 | 127 |
| 8 | LUMACAFTOR | 1782 | -9.4 | 127 |
| 9 | DROSPIRENONE | 855 | -9.3 | 150 |
| 10 | ERGOCALCIFEROL | 10 | -9.2 | 177 |
| 11 | ADAPALENE | 718 | -9.1 | 210 |
| 12 | ALECTINIB | 1819 | -9.1 | 210 |
| 13 | LIFITEGRAST | 1824 | -9.1 | 210 |
| 14 | DIHYDROERGOTAMINE | 36 | -9 | 249 |
| 15 | CYPROHEPTADINE | 303 | -9 | 249 |
| 16 | DUTASTERIDE | 883 | -9 | 249 |
| 17 | M DOXERCALCIFEROL | 1556 | -9 | 249 |
| 18 | MIDOSTAURIN | 1828 | -9 | 249 |
| 19 | M REVEFENACIN | 1910 | -9 | 249 |
| 20 | ESTRADIOL CYPIONATE | 20 | -8.9 | 294 |
| 21 | BROMOCRIPTINE | 470 | -8.9 | 294 |
| 22 | BEXAROTENE | 847 | -8.9 | 294 |
| 23 | CONIVAPTAN | 927 | -8.9 | 294 |
| 24 | BETA CAROTENE | 1380 | -8.9 | 294 |
| 25 | CALCITRIOL | 480 | -8.8 | 349 |
| 26 | SIROLIMUS | 853 | -8.8 | 349 |
| 27 | CANDICIDIN | 1079 | -8.8 | 349 |
| 28 | PARITAPREVIR | 1734 | -8.8 | 349 |
| 29 | M MIDOSTAURIN | 1829 | -8.8 | 349 |
| 30 | M MIDOSTAURIN | 1852 | -8.8 | 349 |
| 31 | MOXIDECTIN | 1916 | -8.8 | 349 |
| 32 | ENTRECTINIB | 1969 | -8.8 | 349 |
| 33 | HEXAFLUORENIUM | 1336 | -8.7 | 413 |
| 34 | ELTROMBOPAG | 1450 | -8.7 | 413 |
| 35 | SIMEPREVIR | 1628 | -8.7 | 413 |
| 36 | VENETOCLAX | 1822 | -8.7 | 413 |
| 37 | M ENTRECTINIB | 1970 | -8.7 | 413 |
| 38 | CAPMATINIB | 1983 | -8.7 | 413 |
| 39 | **QUINESTROL** | 383 | -8.6 | 489 |
| 40 | MARAVIROC | 958 | -8.6 | 489 |
| 41 | NYSTATIN | 990 | -8.6 | 489 |
| 42 | ERGOLOID | 1213 | -8.6 | 489 |
| 43 | CALCIFEDIOL | 1390 | -8.6 | 489 |
| 44 | ABIRATERONE ACETATE | 1519 | -8.6 | 489 |
| 45 | PONATINIB | 1593 | -8.6 | 489 |
| 46 | NETUPITANT | 1726 | -8.6 | 489 |
| 47 | TUCATINIB | 1982 | -8.6 | 489 |
| 48 | LURBINECTEDIN | 1986 | -8.6 | 489 |
| 49 | EXATECAN | 1993 | -8.6 | 489 |
| 50 | PIMOZIDE | 424 | -8.5 | 579 |
| 51 | DANAZOL | 435 | -8.5 | 579 |
| 52 | TELMISARTAN | 814 | -8.5 | 579 |
| 53 | HYDROCORTISONE CYPIONATE | 1327 | -8.5 | 579 |
| 54 | VORAPAXAR | 1710 | -8.5 | 579 |
| 55 | DASABUVIR | 1735 | -8.5 | 579 |
| 56 | ELBASVIR | 1815 | -8.5 | 579 |
| 57 | GLECAPREVIR | 1850 | -8.5 | 579 |
| 58 | AVATROMBOPAG | 1878 | -8.5 | 579 |
| 59 | BALOXAVIR | 1914 | -8.5 | 579 |
| 60 | TRIFAROTENE | 1944 | -8.5 | 579 |
| 61 | AVAPRITINIB | 1968 | -8.5 | 579 |
| 62 | LOPERAMIDE | 450 | -8.4 | 685 |
| 63 | ITRACONAZOLE | 678 | -8.4 | 685 |
| 64 | RISPERIDONE | 713 | -8.4 | 685 |
| 65 | EXEMESTANE | 795 | -8.4 | 685 |
| 66 | CHOLECALCIFEROL | 824 | -8.4 | 685 |
| 67 | DACTINOMYCIN | 1058 | -8.4 | 685 |
| 68 | TESTOSTERONE CYPIONATE | 1335 | -8.4 | 685 |
| 69 | ERIBULIN | 1505 | -8.4 | 685 |
| 70 | RILPIVIRINE | 1516 | -8.4 | 685 |
| 164 | **MESTRANOL** | 213 | -7.9 | 1595 |
| 203 | **ETHINYL ESTRADIOL** | 18 | -7.8 | 1889 |

**Supplementary Figure S1.** **Docking of steroid drugs to *Staphylococcus aureus* cytochrome *bd***. (*A*) Cytochrome *bd*-I CydA structure with highlighted quinol binding site. (*B*) Quinestrol docked in the quinol binding site (*C*) Mestranol docked in the quinol binding site (*D*) Ethinyl estradiol docked in the quinol binding site. Red = negative charge. White = hydrophobic. Blue = positive charge.


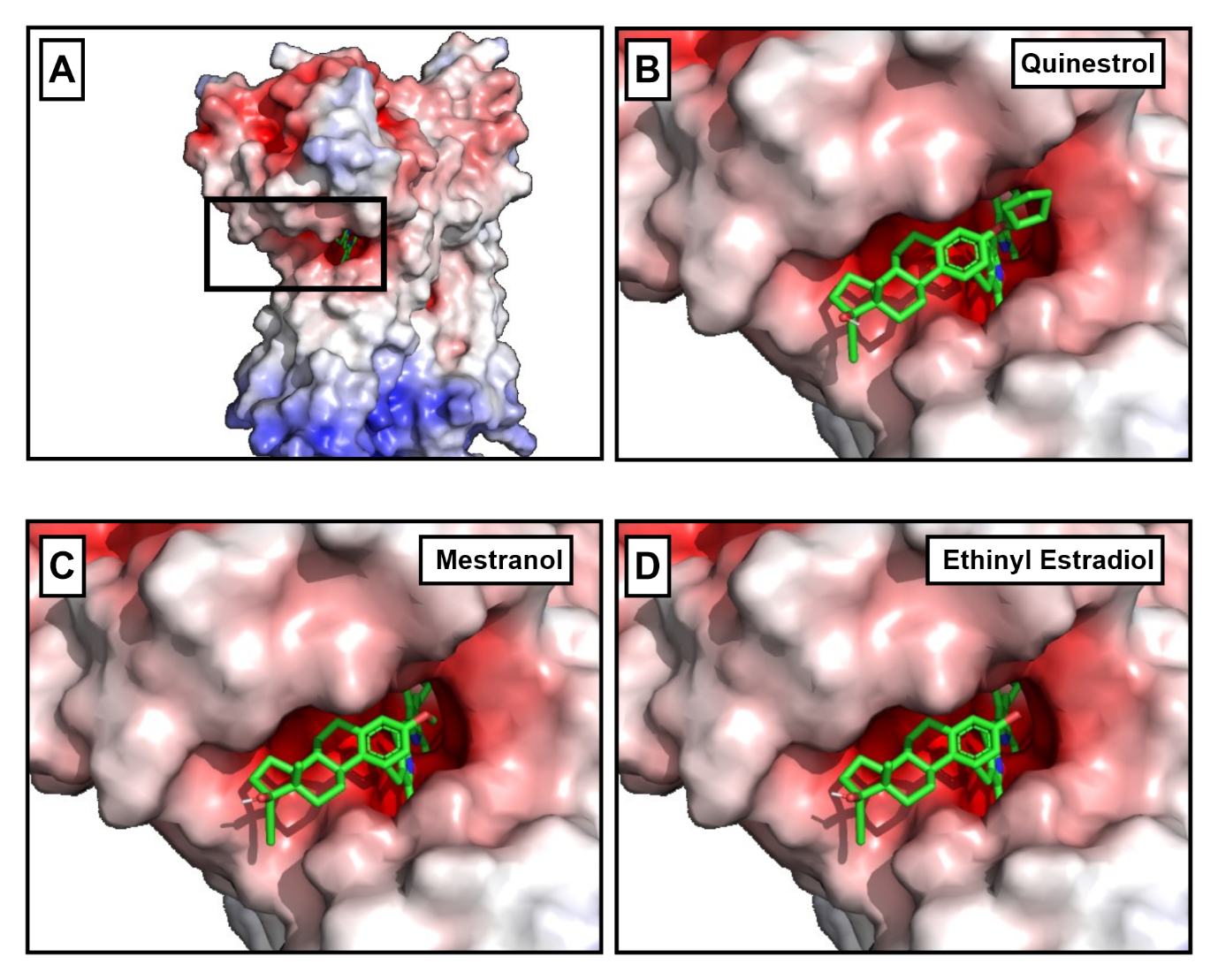


| **Drug Name** | **eDrug3D Database Number** | **Predicted Affinity (kcal/mol)** | **Estimated**  ***K*_d_ (nM)** |
| --- | --- | --- | --- |
| **Quinestrol** | 383 | -8.1 | 1138 |
| **Mestranol** | 213 | -7.5 | 3136 |
| **Ethinyl Estradiol** | 18 | -7.2 | 5206 |

**Supplementary Table S2. Steroid compounds and binding affinities to *S. aureus* CydA quinol binding site**.

**Supplementary Figure S2.** **Raw traces demonstrating oxygen consumption activity in *E. coli* ‘*bd*-only’ membranes**. Linear regression fits were done to calculate the rate of oxygen consumption from timepoint 1000 – 1300 secs.

**Supplementary Figure S3**. **Succinate dehydrogenase (SDH) *E. coli* membrane assay with quinestrol**. The activity of SDH was expressed as a percentage of the DMSO only control. Data represent the means of three repeats and error bars represent the standard deviation.

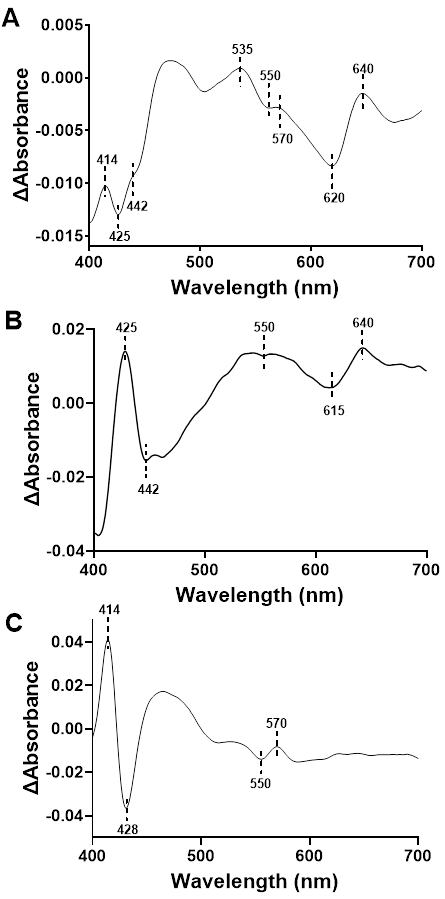
**Supplementary Figure S4**. **CO difference spectra of *E. coli* WT, ‘*bd*-I only’ and *bo*′-only cells**. The figure represents A) *E. coli* WT cells, B) *E. coli ‘bd*-I only’ cells and C) *E. coli ‘bo*′ only’ cells. Cells were reduced using sodium dithionite after which they were exposed to carbon monoxide for 5 min. Reduced spectra were subtracted from cells that were reduced and exposed to carbon monoxide to get CO difference spectra.

**Supplementary Figure S5. Raw traces of *E. coli* ‘*bd*-only’ growth curves in the presence of quinestrol.** µmax is calculated via liners regression of datapoints from the region of most rapid growth (2 - 3 h) and allows direct comparison of all quinestrol concentrations.

**Supplementary Figure S6**. **Quinestrol inhibits oxygen consumption activity of MRSA ‘*bd*-only’ membranes.** The final concentration of membranes in the reaction chamber was 1 mg/mL and the reaction was initiated via the addition of 500 µM NADH as the substrate. Quinestrol exhibited an IC_50_ of 0.98 ± 0.1 µg/mL (2.3 ± 0.2 µM).
